# Supplementary material for: Entropy of a bacterial stress response is a generalizable predictor for fitness and antibiotic sensitivity
Source: Nat Commun. 2020 Aug 31;11:4365. doi: 10.1038/s41467-020-18134-z (PMC7458919; doi:10.1038/s41467-020-18134-z)
Supplement: Supplementary file 1 — Supplementary Information [file 41467_2020_18134_MOESM1_ESM.pdf]

Supplementary Information

Entropy of a bacterial stress response is a generalizable predictor for fitness and antibiotic sensitivity

Zhu et al.

Supplementary Figures

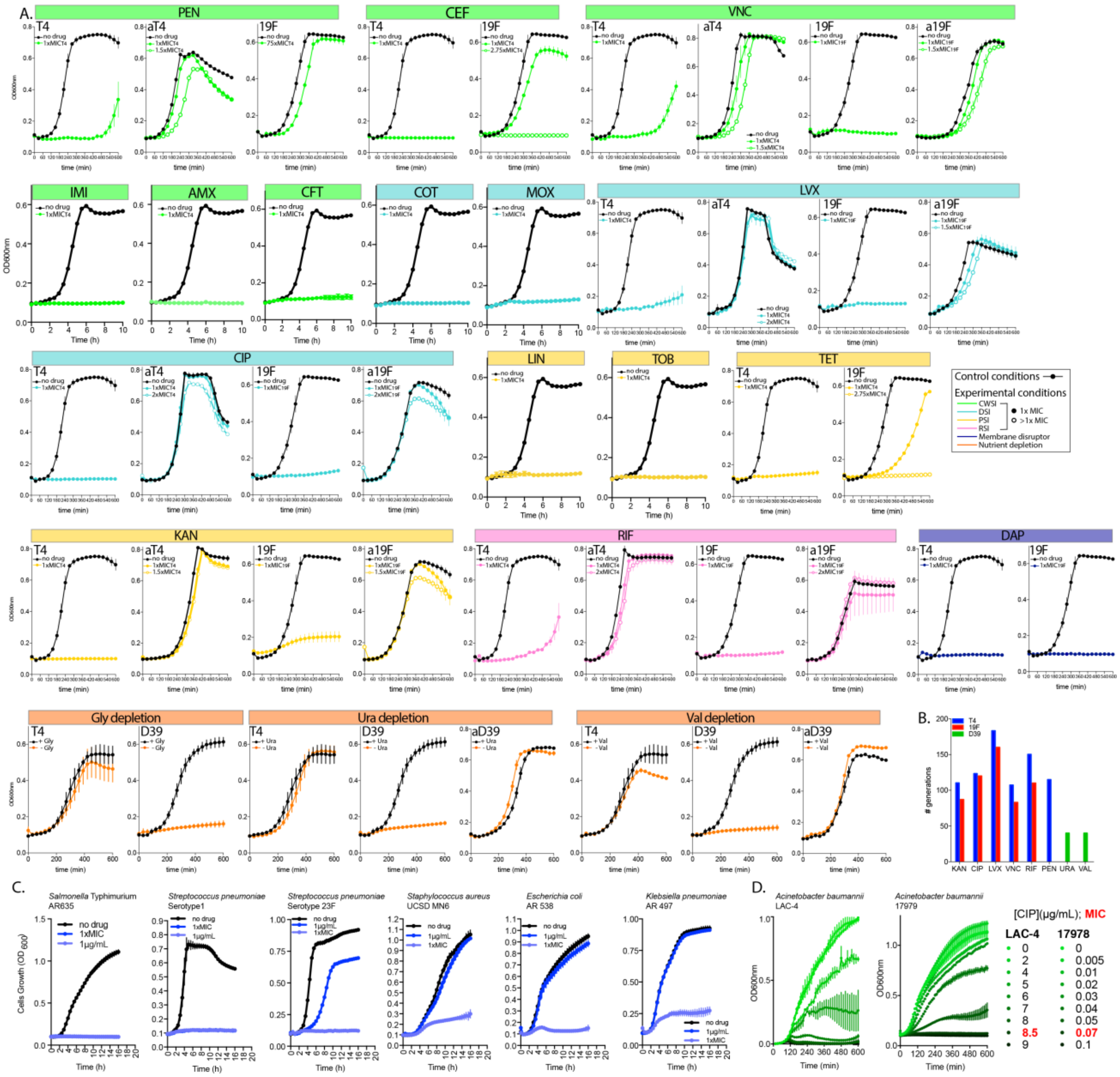

Supplementary Figure 1. High and low fitness outcomes under antibiotic exposure and single nutrient depletion.

A. Growth curves of stress-sensitive *S. pneumoniae* T4 and 19F strains and antibiotic- or nutrient-adapted strains (labeled as aT4, a19F and aD39). **B.** Number of generations of adapted populations. Detailed information on minimum inhibitory concentrations is listed in **Supplementary Table 2**. **C**. Growth curves of *S.* Typhimurium, *S. pneumoniae* serotypes 1 and 23F strains, *S. aureus*, *E. coli* and *K. pneumoniae* under 1μg per mL and strain-specific minimum inhibitory concentration (1xMIC) of ciprofloxacin. **D.** Growth curves of ciprofloxacin MIC determination for *A.baummannii* strains LAC-4 and ATCC 17978. Mean ±standard error of n=3 biological replicates are shown in panels **A**, **C** and **D**.

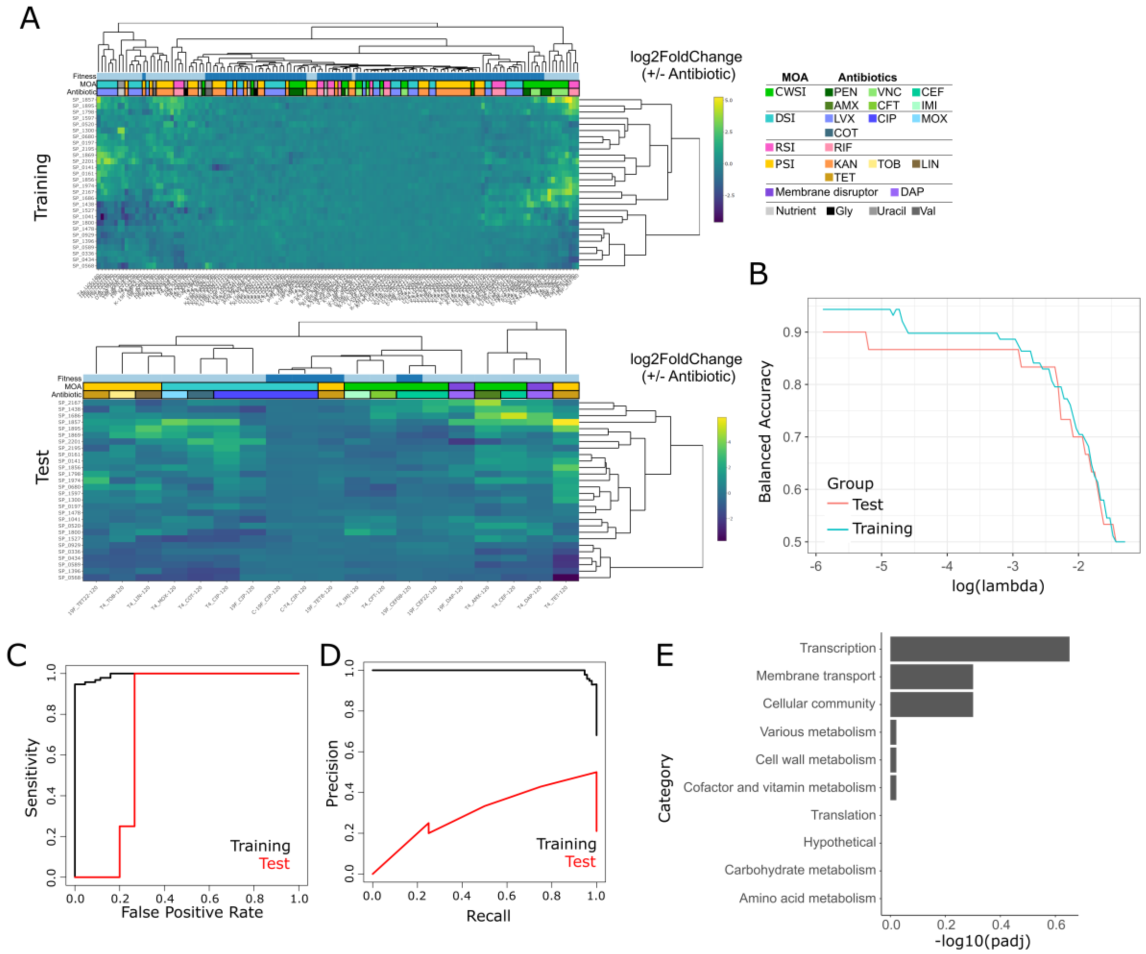

Supplementary Figure 2. Performance and functional enrichment of the gene-panel that predicts fitness.

A. Heatmaps show the differential expression (log2FoldChange) of each gene in the panel in each of the 19 stress conditions. Each row is a gene in the panel, and each column is a different experiment (experimental timepoints are separate columns). Top: Training set data, Bottom: Test set data. The top bar above the heatmap shows the observed fitness outcome (light blue: low fitness, dark blue: high fitness). The middle and bottom bars above the heatmaps indicate the MOA and identity of the stress respectively. Dendrograms on the top and side of the heatmaps show hierarchical clustering of the columns and rows respectively. **B.** Balanced accuracy of the regression model is similar for training and test sets at different values of lambda. For lambda < 0.05, both train and test set accuracies are > 0.85, despite the models selecting different sets of genes (Figure 1E). **C**. Receiver-operator characteristic (ROC) curve for the fitness gene-panel. The area under the curve is 0.99 and 0.75 for the training and test sets respectively. **D**. Precision-Recall (PR) curve for the fitness gene-panel. The area under the curve is 0.99 and 0.31 for the training and test sets respectively. **E.** No functional category is enriched in the fitness gene-panel. For each category present in the gene-panel, a hypergeometric test was performed, and the resulting p-value is adjusted for false discoveries (padj). No category had padj<0.01.

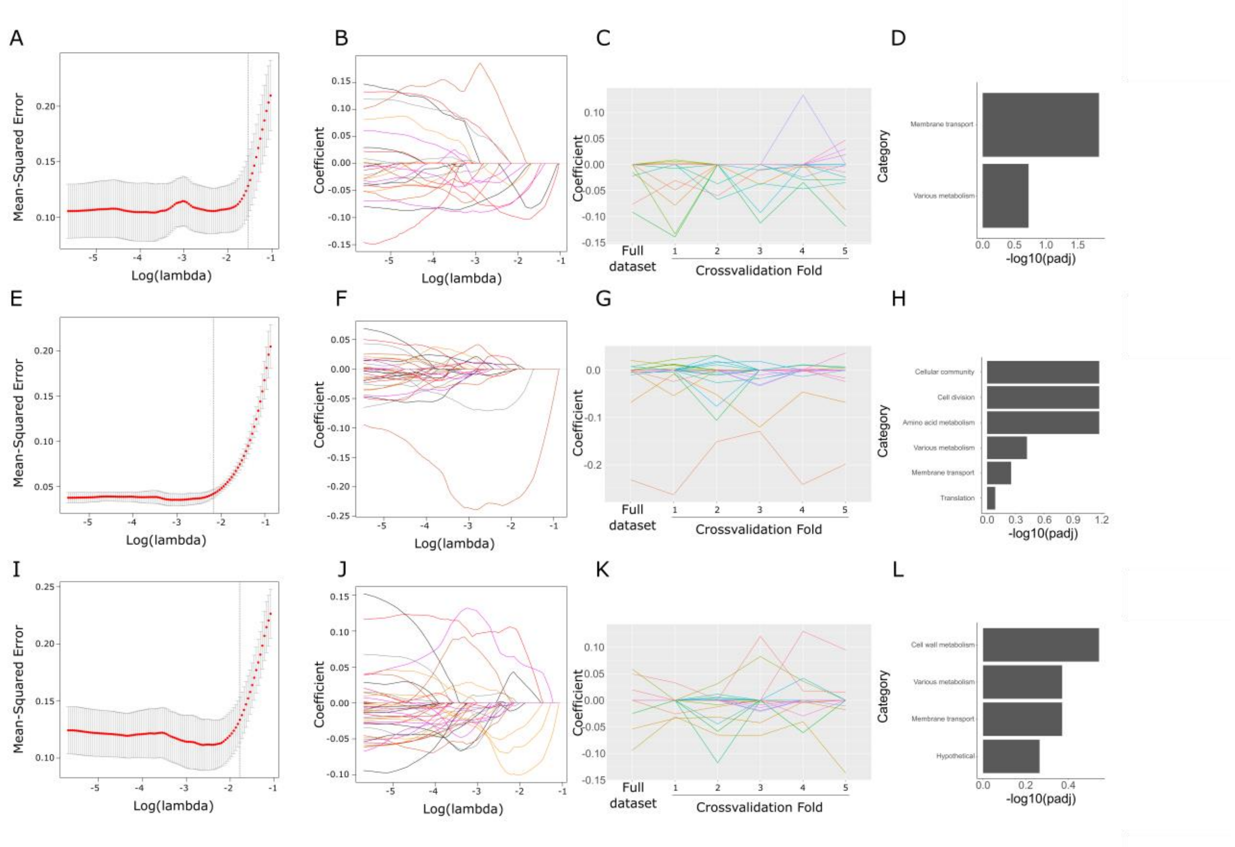

Supplementary Figure 3. Gene-panels that predict fitness for specific MOA's are also sensitive to input data, lambda and show no enrichment.

(**A-D**) CWSI-specific panel. (**E-H**) DSI-specific panel. (**I-L**) PSI-specific panel. **A, E, I** show the crossvalidation analysis that determine the value of lambda (as in Figure 1B). The selected lambda is shown as the dashed line. Red points and error bars represent mean ±standard deviation of error across n=5 crossvalidation folds. **B, F, J** show the coefficients of each gene changing depending on lambda. **C, G, K** show the coefficients of each gene changing with different input data used. Full dataset: coefficients obtained when the regression model is trained on all available training data for a specific MOA. Crossvalidation fold: coefficients obtained when the model is trained on 80% of the available training data. **D, H, L** show enrichment analysis of each gene-panel predicting fitness specific to CWSI, DSI, PSI respectively (similar to Supplemental Figure 2E). There are no functional categories with padj<0.01.

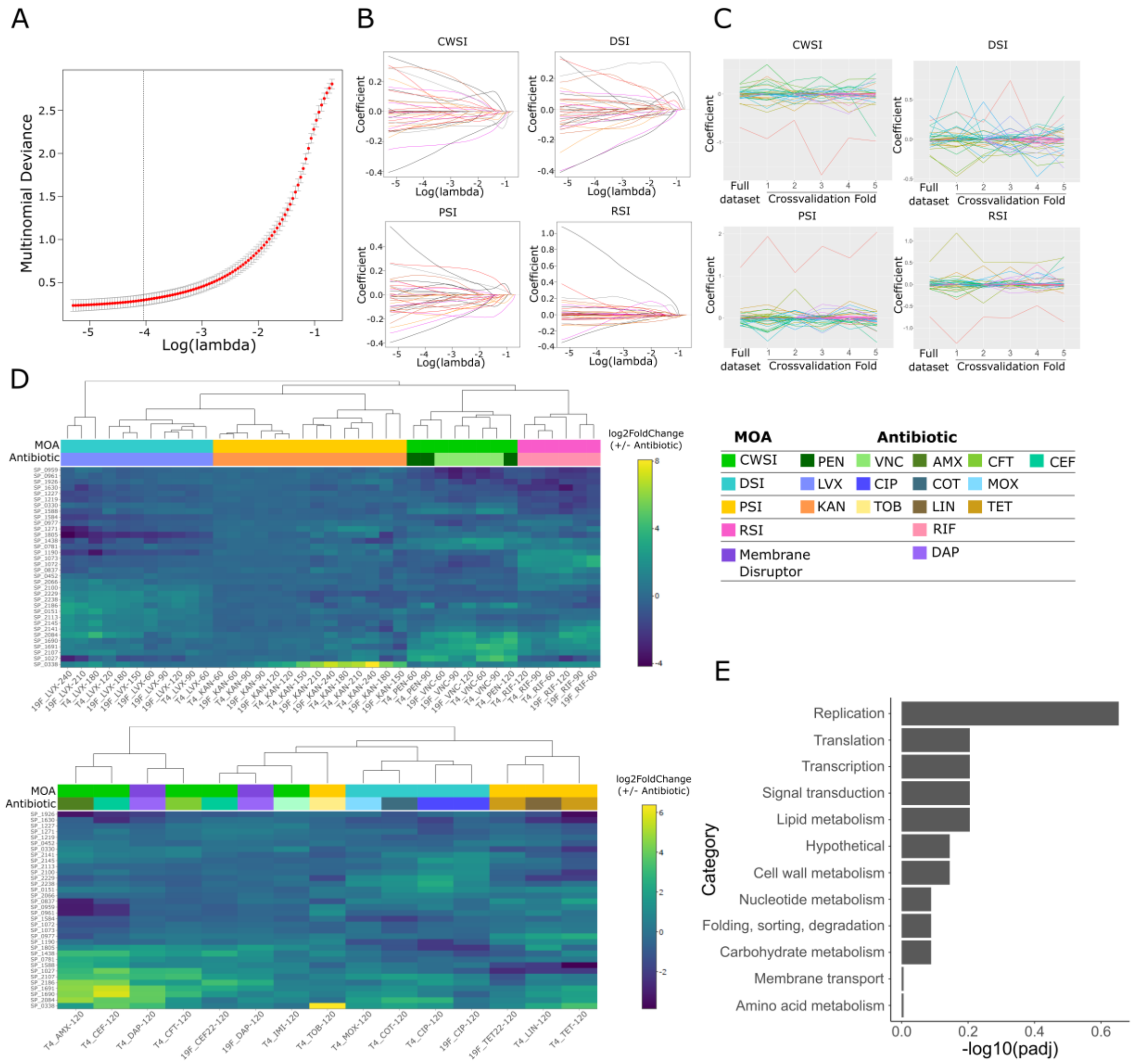

Supplementary Figure 4. Performance of the gene-panel that predicts MOA.
A. Crossvalidation analysis was applied to determine the best value of lambda on the multi-class regression model that predicts MOA. Unlike the 2-class models, error is evaluated as multinomial deviance. Otherwise, lambda is determined the same way as in Figure 1B. Red points and error bars represent mean ±standard deviation of error across n=5 crossvalidation folds. **B.** Coefficients of each gene for each class (i.e. MOA, shown as separate sub-panels) change monotonically as lambda is decreased. This is indicative of the genes being more consistent than the gene-panels that predict fitness (Figure 1E, Supplemental Figure 3B, F, J). **C.** Coefficients of each gene, for each class (sub-panels) are affected by input data. Analysis similar to that done in Figure 1D and Supplemental Figure 3C, G, K. **D.** Heatmaps show differential expression (log2FoldChange) of each gene in the MOA gene-panel (rows) in each experimental condition (columns). The bars directly above the heatmaps show the MOA and the antibiotic. Top panel: training set. Bottom panel: test set. Dendrograms above heatmaps show hierarchical clustering of the experiments. **E.** Functional category enrichment analysis was done similarly to Supplementary Figure 2E. There are no categories with padj<0.01.

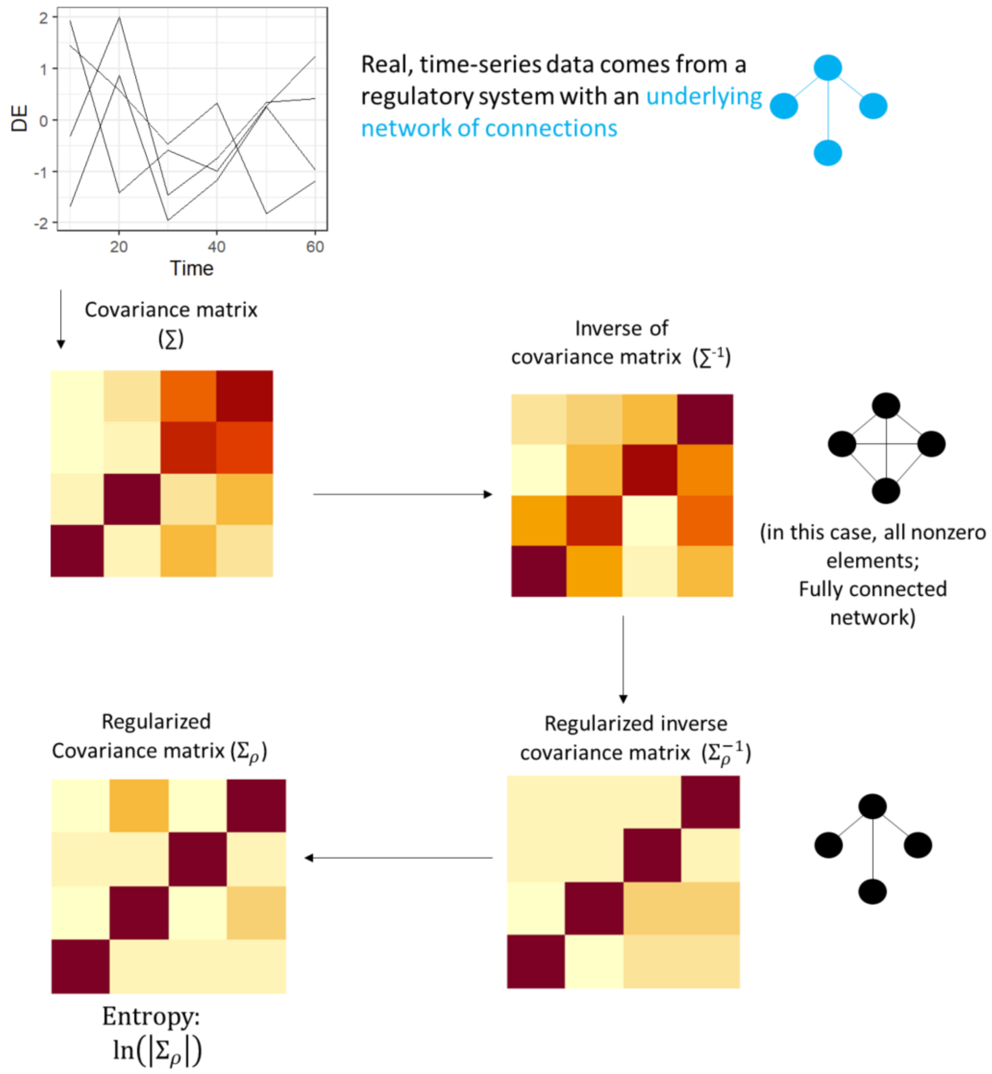

Supplementary Figure 5. Schematic demonstrating how entropy is computed from time-series DE data.

The observable DE patterns are assumed to be influenced by condition-specific networks of interactions among genes. These interactions are unknown, but can be inferred from the covariances among genes. Entropy quantifies disorder on a transcriptome, taking into account these interactions. In order to achieve this, we first compute the covariance matrix (Σ) across genes, and take its inverse (Σ^{-1}). The support of this inverse covariance matrix yields a dense network, which is the "uncorrected" version of the real coexpression network. Since the real network is assumed to be sparse, we apply graphical lasso to retrieve a sparse network $\Sigma_\rho{}^{-1}$, and invert the resulting matrix (Σ_ρ). Entropy is defined as the logarithm of the determinant of this matrix.

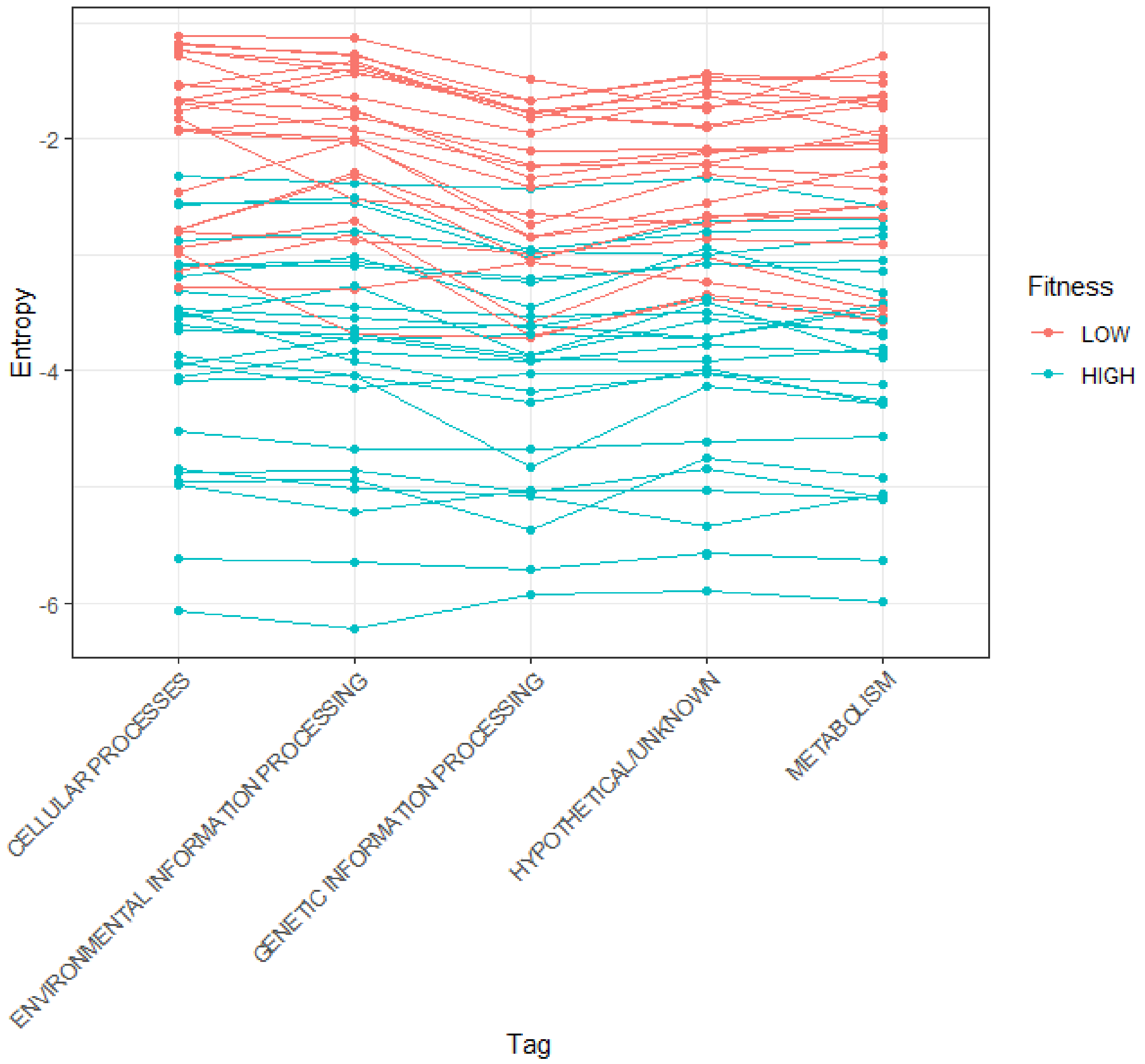

Supplementary Figure 6. Entropy for different functions is similar.
Each of the 55 experiments is represented by a line. Temporal entropy with $\rho=\infty$ was computed on each of the 5 sets of genes belonging to different functions was computed. The entropy values computed on the 5 non-overlapping gene sets separated by functional tags are similar for each experiment.

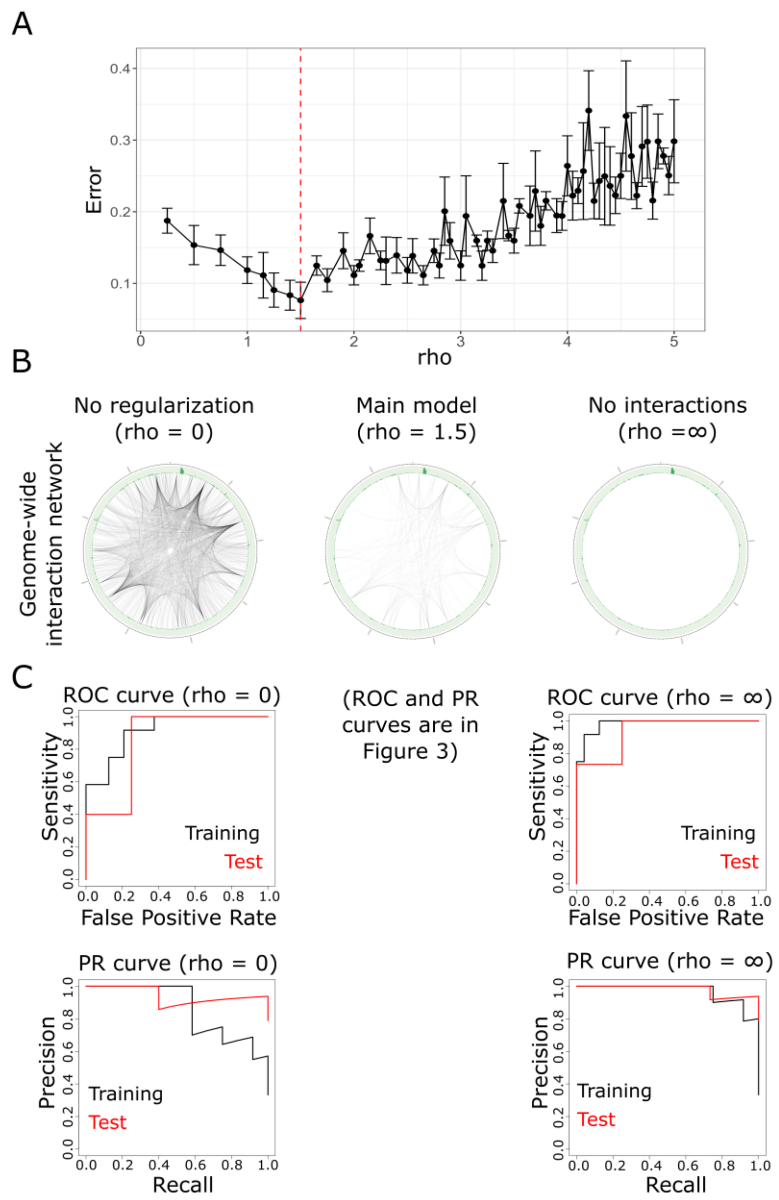

Supplementary Figure 7. Variants of entropy on time course data also predict fitness with high performance.

A. (Same as Main Figure 3C) To test whether entropy was sensitive to regularization parameter rho, two extreme values of rho were used, as opposed to the optimal value of rho=1.5 determined based on crossvalidation error. Black points and error bars represent mean ± standard deviation of error across n=5 crossvalidation folds. **B.** For rho=0 (corresponding to no regularization, and a dense network of gene-to-gene interactions), and for rho = ∞ (corresponding to no interactions, and an empty network), the resulting networks for wildtype T4 exposed to VNC are shown. **C.** These two extreme models were evaluated using ROC and PR curves, and resulted in areas under the curve ≥0.85 for all cases in both PR and ROC, for training and test datasets. Full list of performance statistics can be found in Supplemental Table 8.

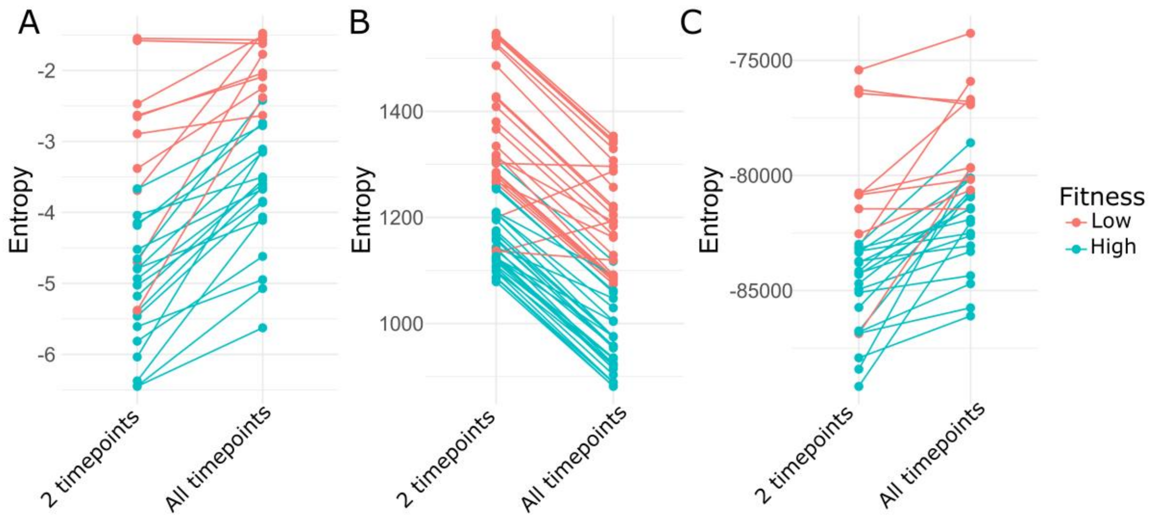

Supplementary Figure 8. Entropy is affected by the number of timepoints used.
For each of the temporal models, corresponding to ρ=∞ (**A**), ρ=1.5 (**B**), and ρ=0 (**C**), entropy was computed from the same 2 timepoints (30 and 120 minutes), or alternatively all timepoints available (which can be up to 11 timepoints). The same experiment is connected by a line. For all 3 variants of temporal entropy, the change in the direction when using all timepoints is consistent across experiments.

Supplementary Tables

Supplementary Table 1. Project setup

Experimental Setup				Data Collection	Predictions			
**Stress**	**Species**	**Strain**	**Fitness**	**RNA-Seq timepoints (min)**	**Fitness (Gene-panel)**	**MOA (Gene-panel)**	**Entropy**	**Entropy(single timepoint)**
Amoxicillin	*Streptococcus pneumoniae*	T4	Low	30, 120	Test	Test	Test	Test
Cefepime	*Streptococcus pneumoniae*	T4	Low	30, 120	Test	Test	Test	Test
Cefepime	*Streptococcus pneumoniae*	19F	Low, High	30, 120	Test	Test	Test	Test
Ceftriaxone	*Streptococcus pneumoniae*	T4	Low	30, 120	Test	Test	Test	Test
Ciprofloxacin	*Streptococcus pneumoniae*	T4	Low	30, 120	Test	Test	Test	Test
Ciprofloxacin	*Streptococcus pneumoniae*	19F	Low	30, 120	Test	Test	Test	Test
Ciprofloxacin	*Streptococcus pneumoniae*	aT4	High	30, 120	Test		Test	Test
Ciprofloxacin	*Streptococcus pneumoniae*	a19F	High	30, 120	Test		Test	Test
Ciprofloxacin	*Streptococcus pneumoniae*	23F	Low, High	120				Validation
Ciprofloxacin	*Streptococcus pneumoniae*	1	Low	120				Validation
Ciprofloxacin	*Salmonella* Typhimurium	AR635 (CDC)	Low	120				Validation
Ciprofloxacin	*Staphylococcus aureus*	MN6 (CDC)	Low, High	120				Validation
Ciprofloxacin	*Escherichia coli*	AR538 (CDC)	Low, High	120				Validation
Ciprofloxacin	*Klebsiella pneumoniae*	AR497 (CDC)	Low, High	120				Validation
Ciprofloxacin	*Acinetobacter baumannii*	LAC-4	High	120				Validation
Ciprofloxacin	*Acinetobacter baumannii*	17978	Low	120				Validation
Cotrimoxazol	*Streptococcus pneumoniae*	T4	Low	30, 120	Test	Test	Test	Test
Daptomycin	*Streptococcus pneumoniae*	T4	Low	30, 120	Test		Test	Test
Daptomycin	*Streptococcus pneumoniae*	19F	Low	30, 120	Test		Test	Test
Imipenem	*Streptococcus pneumoniae*	T4	Low	30, 120	Test	Test	Test	Test
Kanamycin	*Streptococcus pneumoniae*	T4	Low	10, 20, 30, 45, 60, 90, 120, 150, 180, 210, 240	Training	Training	Training	Training
Kanamycin	*Streptococcus pneumoniae*	19F	Low	10, 20, 30, 45, 60, 90, 120, 150, 180, 210, 240	Training	Training	Training	Training
Kanamycin	*Streptococcus pneumoniae*	aT4	High	30, 60, 90, 120, 150, 180, 210, 240	Training		Training	Training
Kanamycin	*Streptococcus pneumoniae*	a19F	High	30, 60, 90, 120, 150, 180, 210, 240	Training		Training	Training
Levofloxacin	*Streptococcus pneumoniae*	T4	Low	10, 20, 30, 45, 60, 90, 120, 150, 180	Training	Training	Training	Training
Levofloxacin	*Streptococcus pneumoniae*	19F	Low	30, 60, 90, 120, 150, 180, 210, 240	Training	Training	Training	Training
Levofloxacin	*Streptococcus pneumoniae*	aT4	High	30, 60, 90, 120, 150, 180, 210, 240	Training		Training	Training
Levofloxacin	*Streptococcus pneumoniae*	a19F	High	30, 60, 90, 120, 150, 180, 210, 240	Training		Training	Training
Linezolid	*Streptococcus pneumoniae*	T4	Low	30, 120	Test	Test	Test	Test
Moxifloxacin	*Streptococcus pneumoniae*	T4	Low	30, 120	Test	Test	Test	Test
No Glycine	*Streptococcus pneumoniae*	T4	High	30, 90	Training		Training	Training
No Glycine	*Streptococcus pneumoniae*	D39	Low	30, 90	Training		Training	Training
No Uracil	*Streptococcus pneumoniae*	T4	High	30, 90	Training		Training	Training
No Uracil	*Streptococcus pneumoniae*	D39	Low	30, 60, 90, 120	Training		Training	Training
No Uracil	*Streptococcus pneumoniae*	aD39	High	30, 90	Training		Training	Training
No Valine	*Streptococcus pneumoniae*	T4	High	30, 90	Training		Training	Training
No Valine	*Streptococcus pneumoniae*	D39	Low	30, 90	Training		Training	Training
No Valine	*Streptococcus pneumoniae*	aD39	High	30, 20	Training		Training	Training
Penicillin	*Streptococcus pneumoniae*	T4	Low	10, 20, 30, 45, 60, 90, 120	Training	Training	Training	Training
Penicillin	*Streptococcus pneumoniae*	19F	High	10, 20, 30, 45, 60, 90, 120	Training		Training	Training
Penicillin	*Streptococcus pneumoniae*	aT4	High	10, 20, 30, 45, 60, 90, 120	Training		Training	Training
Rifampicin	*Streptococcus pneumoniae*	T4	Low	10, 20, 30, 45, 60, 90, 120	Training	Training	Training	Training
Rifampicin	*Streptococcus pneumoniae*	19F	Low	10, 20, 30, 45, 60, 90, 120	Training	Training	Training	Training
Rifampicin	*Streptococcus pneumoniae*	aT4	High	10, 20, 30, 45, 60, 90, 120	Training		Training	Training
Rifampicin	*Streptococcus pneumoniae*	a19F	High	10, 20, 30, 45, 60, 90, 120	Training		Training	Training
Tetracycline	*Streptococcus pneumoniae*	T4	Low	30, 120	Test	Test	Test	Test
Tetracycline	*Streptococcus pneumoniae*	19F	Low, High	30, 120	Test	Test	Test	Test
Tobramycin	*Streptococcus pneumoniae*	T4	Low	30, 120	Test	Test	Test	Test
Vancomycin	*Streptococcus pneumoniae*	T4	Low	10, 20, 30, 45, 60, 90	Training	Training	Training	Training
Vancomycin	*Streptococcus pneumoniae*	19F	Low	10, 20, 30, 45, 60, 90, 120	Training	Training	Training	Training
Vancomycin	*Streptococcus pneumoniae*	aT4	High	10, 20, 30, 45, 60, 90, 120	Training		Training	Training
Vancomycin	*Streptococcus pneumoniae*	a19F	High	10, 20, 30, 45, 60, 90, 120	Training		Training	Training

Cell Wall Synthesis Inhibitor
DNA Synthesis Inhibitor
Membrane Disruptor
Protein Synthesis Inhibitor
Nutrient Depletion
RNA Synthesis Inhibitor

Supplementary Table 2. Antibiotic minimum inhibitory concentrations (MIC) used in this study

Antibiotic	1x MIC for T4 (µg per mL)	1x MIC for 19F (µg per mL)
Amoxicilin (AMX)	0.12	na
Cefepime (CEF)	0.8	2.2
Ceftriaxone (CFT)	0.007	na
Ciprofloxacin (CIP)	1	1
Cotrimoxazol (COT)	6.5	na
Daptomycin (DAP)	35	35
Imipenem (IMI)	0.03	na
Kanamycin (KAN)	90	90
Levofloxacin (LVX)	1	1.1
Linezolid (LIN)	0.5	na
Moxifloxacin (MOX)	0.45	na
Penicillin (PEN)	0.03	2.25
Rifampicin (RIF)	0.035	0.035
Tetracycline (TET)	8	22
Tobramycin (TOB)	175	na
Vancomycin (VNC)	0.24	0.24

na: not tested in this study.

Supplementary Table 3: Features and their coefficients in the fitness gene panel. Feature: the TIGR4 locus tag of the selected feature. Coefficient: the coefficient of the feature. Tag: Functional tag, Category: Functional category of the gene.

Feature	Coefficient	Tag	Category	Gene Name	Gene Description
**SP_0161**	-0.010279271	GENETIC INFORMATION PROCESSING	Transcription		
**SP_0568**	0.025001143	GENETIC INFORMATION PROCESSING	Translation		valyl-tRNA synthetase
**SP_1300**	-0.034269552	HYPOTHETICAL/UNKNOWN	NA		Hypothetical Protein
**SP_1869**	-0.03957627	ENVIRONMENTAL INFORMATION PROCESSING	Membrane transport		
**SP_2195**	-0.019990526	GENETIC INFORMATION PROCESSING	Transcription	*ctsR*	Transcriptional regulator CtsR
**SP_0929**	0.032539524	GENETIC INFORMATION PROCESSING	Translation	*rluD*	Ribosomal large subunit pseudouridine synthase D
**SP_0141**	-0.00392369	GENETIC INFORMATION PROCESSING	Transcription		transcriptional regulator, MutR family
**SP_0680**	-0.019730034	GENETIC INFORMATION PROCESSING	Translation	*rsuA-2*	Ribosomal small subunit pseudouridine synthase A
**SP_0197**	-0.00602077	METABOLISM	Cofactor and vitamin metabolism		Dihydrofolate synthase (EC 6.3.2.12)
**SP_0336**	0.022187461	METABOLISM	Cell wall metabolism		Penicillin-binding protein 2x
**SP_1438**	0.009735969	ENVIRONMENTAL INFORMATION PROCESSING	Membrane transport		ABC transporter, ATPase component
**SP_0434**	0.024817121	HYPOTHETICAL/UNKNOWN	NA		
**SP_0520**	-0.023400459	HYPOTHETICAL/UNKNOWN	NA		
**SP_1041**	0.019562441	HYPOTHETICAL/UNKNOWN	NA		
**SP_0589**	0.126448171	METABOLISM	Amino acid metabolism	*cysE*	serine acetyltransferase
**SP_1686**	-0.017175824	HYPOTHETICAL/UNKNOWN	NA		Hypothetical Protein
**SP_1895**	-0.001292326	ENVIRONMENTAL INFORMATION PROCESSING	Membrane transport	*rafG*	sugar ABC transporter substrate-binding protein
**SP_2167**	-0.003983055	METABOLISM	Carbohydrate metabolism	*fucK*	L-fuculose kinase fucK
**SP_1798**	-0.017352287	ENVIRONMENTAL INFORMATION PROCESSING	Membrane transport		
**SP_1800**	0.093566147	GENETIC INFORMATION PROCESSING	Transcription		
**SP_1856**	-0.08663485	GENETIC INFORMATION PROCESSING	Transcription		
**SP_1857**	-0.008283306	ENVIRONMENTAL INFORMATION PROCESSING	Membrane transport		Cation efflux system protein
**SP_1396**	0.000580467	ENVIRONMENTAL INFORMATION PROCESSING	Membrane transport	*pstB1*	Phosphate ABC transporter, ATP-binding component I
**SP_1478**	0.029056712	METABOLISM	Various metabolism		Aldo/keto reductase
**SP_1527**	0.006450843	ENVIRONMENTAL INFORMATION PROCESSING	Membrane transport	*aliB*	Oligopeptide ABC transporter, oligopeptide-binding protein
**SP_1597**	-0.015615773	HYPOTHETICAL/UNKNOWN	NA		Hypothetical Protein
**SP_2201**	-0.040685009	CELLULAR PROCESSES	Cellular community	*cbpD*	Late competence protein cbpD - murein hydrolase
**SP_1974**	-0.016625727	METABOLISM	Various metabolism		Acylphosphatase
**(Intercept)**	0.760173069	NA	NA	NA	NA

Supplementary Table 4: Features and their coefficients in the MOA-specific fitness panels. Panel: which panel the feature belongs to. Feature: the TIGR4 locus tag of the selected feature. Coefficient: the coefficient of the feature. Tag: Functional tag, Category: Functional category of the gene.

Panel	Feature	Coefficient	Tag	Category	Gene Name	Gene Description
**CWSI-specific fitness**	(Intercept)	0.802510556				
**CWSI-specific fitness**	SP_0091	- 0.076552298	ENVIRONMENTAL INFORMATION PROCESSING	Membrane transport	*ugpE*	ABC transporter permease
**CWSI-specific fitness**	SP_0410	- 0.014990773	ENVIRONMENTAL INFORMATION PROCESSING	Membrane transport	NA	exfoliative toxin
**CWSI-specific fitness**	SP_1974	- 0.091374816	METABOLISM	Various metabolism	NA	Acylphosphatase
**CWSI-specific fitness**	SP_2051	- 0.021830947	ENVIRONMENTAL INFORMATION PROCESSING	Membrane transport	*cglC*	Late competence protein ComGC
**DSI-specific fitness**	(Intercept)	0.852083322				
**DSI-specific fitness**	SP_0079	- 0.231416616	ENVIRONMENTAL INFORMATION PROCESSING	Membrane transport	*trkA*	Trk family potassium uptake protein
**DSI-specific fitness**	SP_0106	- 0.068270673	METABOLISM	Amino acid metabolism	*sdaB*	L-serine dehydratase, iron-sulfur-dependent, beta subunit (EC 4.3.1.17)
**DSI-specific fitness**	SP_0894	0.020592366	METABOLISM	Amino acid metabolism	*pepX*	x-prolyl-dipeptidyl aminopeptidase
**DSI-specific fitness**	SP_1398	0.007518056	ENVIRONMENTAL INFORMATION PROCESSING	Membrane transport	*pstA*	Phosphate ABC transporter, permease component
**DSI-specific fitness**	SP_1472	0.007183581	METABOLISM	Various metabolism	NA	Oxidoreductase
**DSI-specific fitness**	SP_2103	-0.00451615	GENETIC INFORMATION PROCESSING	Translation	*rrmA*	23S rRNA (guanine(745)-N(1))-methyltransferase
**DSI-specific fitness**	SP_2201	- 0.005319221	CELLULAR PROCESSES	Cellular community	*cbpD*	Late competence protein cbpD - murein hydrolase
**DSI-specific fitness**	SP_2217	- 0.009811251	CELLULAR PROCESSES	Cell division	*mreD*	rod shpae-determining protein
**PSI-specific fitness**	(Intercept)	0.760960208				
**PSI-specific fitness**	SP_0434	0.0496811	HYPOTHETICAL/UNKNOWN			
**PSI-specific fitness**	SP_0678	0.05860058	HYPOTHETICAL/UNKNOWN			hypothetical protein
**PSI-specific fitness**	SP_0913	- 0.024961142	ENVIRONMENTAL INFORMATION PROCESSING	Membrane transport		ABC transporter permease
**PSI-specific fitness**	SP_0953	- 0.053737175	METABOLISM	Various metabolism		Acetyltransferase
**PSI-specific fitness**	SP_1118	0.019527231	METABOLISM	Cell wall metabolism		pullulanase;
**PSI-specific fitness**	SP_1857	- 0.094105937	ENVIRONMENTAL INFORMATION PROCESSING	Membrane transport		Cation efflux system protein

Supplementary Table 5. Homologs in 6 pathogenic species of genes in 2 gene-panels that predict fitness (corresponding to Figure 1F, G). Homology data was obtained from the PATRIC database[1]. When present, the homolog is represented by its locus tag. An empty cell indicates absence of the homolog in that species. The two fitness panels included are the condition-agnostic gene-panel presented in this work, and the *E. coli* specific CIP gene-panel in a previous study[2].

Gene-panel	PATRIC cross-genus families (PGfams)	*Acinetobacter baumannii* ATCC 17978	*Escherichia coli* str. K-12 substr. MG1655	*Klebsiella pneumoniae* HS11286	*Salmonella enterica* serovar Typhimurium str. LT2	*Staphylococcus aureus* subsp. aureus NCTC 8325	*Streptococcus pneumoniae* TIGR4
**This work**	PGF_00004652		b4490	KPHS_19230		SAOUHSC_00327	SP_1300
**This work**	PGF_00013324						SP_0520
**This work**	PGF_00015320						SP_1869
**This work**	PGF_00018135						SP_1800
**This work**	PGF_00035026						SP_1115
**This work**	PGF_00057506	A1S_1043			STM0354	SAOUHSC_02461	SP_1856
**This work**	PGF_00300574						SP_1041
**This work**	PGF_00402268					SAOUHSC_01907	SP_1478
**This work**	PGF_00417739						SP_2201
**This work**	PGF_01264994		b4280		STM1133		SP_1325
**This work**	PGF_02147779		b1243	KPHS_30000	STM1746.S	SAOUHSC_00927	SP_1527
**This work**	PGF_02640939						SP_2167
**This work**	PGF_03065340					SAOUHSC_02645	SP_0161
**This work**	PGF_03285992						SP_1438
**This work**	PGF_03515040						SP_1597
**This work**	PGF_03520500						SP_1798
**This work**	PGF_03889881					SAOUHSC_00502	SP_2195
**This work**	PGF_04485073	A1S_2868	b2315	KPHS_37740	STM2365	SAOUHSC_01766	SP_0197
**This work**	PGF_04695681	A1S_1407	b3607	KPHS_51110	STM3699	SAOUHSC_00510	SP_0589
**This work**	PGF_05500127	A1S_2742	b4258	KPHS_05160	STM4475	SAOUHSC_01767	SP_0568
**This work**	PGF_06213055	A1S_2445	b3725	KPHS_52970	STM3854	SAOUHSC_01385	SP_1396
**This work**	PGF_06874321						SP_1895
**This work**	PGF_07619772	A1S_1045					SP_1857
**This work**	PGF_09626318	A1S_2120	b2183	KPHS_36820	STM2222	SAOUHSC_01870	SP_0280
**This work**	PGF_10302926	A1S_3204	b0084	KPHS_33530	STM0122	SAOUHSC_01145	SP_1673
**This work**	PGF_10367439		b0968	KPHS_18770	STM1083	SAOUHSC_01406	SP_1974
**This work**	PGF_10569727	A1S_0841	b2594	KPHS_39870	STM2662	SAOUHSC_01163	SP_0929
**This work**	PGF_12783997						SP_0434
**Barczak et al.**	PGF_00026615	A1S_0888	b3959	KPHS_01010	STM4122	SAOUHSC_00147	
**Barczak et al.**	PGF_00047078	A1S_1962	b2699	KPHS_41020	STM2829	SAOUHSC_01262	SP_1940
**Barczak et al.**	PGF_00403095	A1S_0765	b2498	KPHS_38940	STM2498	SAOUHSC_02353	SP_0745
**Barczak et al.**	PGF_00690318		b3645	KPHS_52620			
**Barczak et al.**	PGF_04041316	A1S_3295	b4058	KPHS_02820	STM4254	SAOUHSC_00780	SP_0186

Supplementary References

1. Wattam, A. R. *et al.* PATRIC, the bacterial bioinformatics database and analysis resource. *Nucleic Acids Res* **42**, D581–D591 (2014).
2. Barczak, A. K. *et al.* RNA signatures allow rapid identification of pathogens and antibiotic susceptibilities. *Proc Natl Acad Sci U S A* **109**, 6217–6222 (2012).
