# Supplementary material for: Entropy of a bacterial stress response is a generalizable predictor for fitness and antibiotic sensitivity
Source: Nat Commun. 2020 Aug 31;11:4365. doi: 10.1038/s41467-020-18134-z (PMC7458919; doi:10.1038/s41467-020-18134-z)
Supplement: Supplementary file 3 — Description of Additional Supplementary Files [file 41467_2020_18134_MOESM3_ESM.pdf]

Description of Additional Supplementary Files

Title: Supplementary Data 1

Description: Differential expression (DE) for all experimental conditions included in the training and test datasets. Locus tags (both old and new RefSeq annotation) of the genes in 3 strains (TIGR4, 19F, and D39) are listed in columns A-F. Columns G-P contain functional annotation of all genes. Columns QJY contain differential expression values for each experiment, at each timepoint. The columns are labeled as "[Strain]_[Condition].[Timepoint (min)].DE"

Title: Supplementary Data 2

Description: Enrichment analysis of gene panels presented in Bhattacharyya et al., 2019. For each of the 9 species-antibiotic pairs, the 10-gene panel (from the 2nd phase of model training) was used. Enrichment analysis was performed using a hypergeometric test with Benjamini-Hochberg correction for false discovery. GOterm: name and identifier of the GO term. Category: GO term category (BP: biological process, CC: cellular component, MF: molecular function). pval: unadjusted p-value from hypergeometric test. padj: BH-adjusted p-value. Species: Ab: Acinetobacter baumannii, Ec: Escherichia coli, Kp: Klebsiella pneumoniae. Antibiotic: CIP: ciprofloxacin, GEN: gentamicin, MER: meropenem.

Title: Supplementary Data 3

Description: Predictions using the single-timepoint variant of entropy. Strain: strain background used. Adapted: whether the strain has been adapted to the condition. AB: antibiotic (or nutrient) condition. Concentration: concentration of antibiotic used (L=low, H=high). Time: timepoint in minutes. Survive: real survival outcome. Group: whether the experiment is in the training set or test set. Entropy: value of entropy. Prediction: prediction on survival at this timepoint.

Title: Supplementary Data 4

Description: Features and their coefficients in the MOA gene panel. Feature: the TIGR4 locus tag of the selected feature. Coefficient: the coefficient of the feature. Tag: Functional tag, Category: Functional category of the gene.

Title: Supplementary Data 5

Description: Temporal entropy model predictions. Survive: real survival outcome. Strain: strain background used. Adapted: whether the strain has been adapted to the condition. AB: antibiotic (or nutrient) condition. Concentration: concentration of antibiotic used (L=low, H=high). Group: whether

the experiment is in the training set or test set. For each of the temporal models (Regularization value rho=1.5, ∞, or 0), the entropy values and prediction are listed.

Title: Supplementary Data 6

Description: Model performance for all finalized fitness prediction models. Model: name of the model. Group: training or test set. Number: number of data points in the group. NIR: no information rate, which is the same as the prevalence of the majority outcome. TP: number of true positives. TN: number of true Boston College Department of Biology Higgins Hall, 420A, 140 Commonwealth Avenue, Chestnut Hill, Massachusetts 02467 tel: 617–552–0804 | email: vanopijn@bc.edu | web: vanopijnenlab.com negatives. FP: number of false positives. FN: number of false negatives. PPV: positive predictive value. NPV: negative predictive value.
