# Supplementary material for: Entropy of a bacterial stress response is a generalizable predictor for fitness and antibiotic sensitivity
Source: Nat Commun. 2020 Aug 31;11:4365. doi: 10.1038/s41467-020-18134-z (PMC7458919; doi:10.1038/s41467-020-18134-z)
Supplement: Supplementary file 10 — Reporting Summary [file 41467_2020_18134_MOESM10_ESM.pdf]

nature research

Corresponding author(s): Tim van Opijnen

Last updated by author(s): Jul 22, 2020

Reporting Summary

Nature Research wishes to improve the reproducibility of the work that we publish. This form provides structure for consistency and transparency in reporting. For further information on Nature Research policies, see Authors & Referees and the Editorial Policy Checklist .

Statistics

For all statistical analyses, confirm that the following items are present in the figure legend, table legend, main text, or Methods section.

n/a	Confirmed	
☐	☒	The exact sample size (*n*) for each experimental group/condition, given as a discrete number and unit of measurement
☐	☒	A statement on whether measurements were taken from distinct samples or whether the same sample was measured repeatedly
☐	☒	The statistical test(s) used AND whether they are one- or two-sided<br>*Only common tests should be described solely by name; describe more complex techniques in the Methods section.*
☐	☒	A description of all covariates tested
☐	☒	A description of any assumptions or corrections, such as tests of normality and adjustment for multiple comparisons
☐	☒	A full description of the statistical parameters including central tendency (e.g. means) or other basic estimates (e.g. regression coefficient) AND variation (e.g. standard deviation) or associated estimates of uncertainty (e.g. confidence intervals)
☐	☒	For null hypothesis testing, the test statistic (e.g. *F*, *t*, *r*) with confidence intervals, effect sizes, degrees of freedom and *P* value noted<br>*Give P values as exact values whenever suitable.*
☒	☐	For Bayesian analysis, information on the choice of priors and Markov chain Monte Carlo settings
☒	☐	For hierarchical and complex designs, identification of the appropriate level for tests and full reporting of outcomes
☒	☐	Estimates of effect sizes (e.g. Cohen's *d*, Pearson's *r*), indicating how they were calculated

Our web collection on statistics for biologists contains articles on many of the points above.

Software and code

Policy information about availability of computer code

Data collection	RNA-Seq data were generated using the BaseSpace bcl2fastq (v2.19) software by Illumina
Data analysis	RNA-Seq data were processed using aerobio (https://github.com/jsa-aerial/aerobio). This processing includes bowtie2 (v2.2.6) for alignment, and DESeq2 (v1.10.1) for differential expression analysis. All further analyses were done using R (v3.6.2) and MATLAB (R2016b). Logistic regression analyses were done using the package glmnet (v.3.0-2). For data processing and visualization, plotmo (v3.5.6), heatmaply (v1.0), caret (v6.0-85), PRROC (v1.3.1) and ggplot2(v3.2.1) were used. The custom code used in this manuscript can be found at https://github.com/dsurujon/FitnessPrediction

For manuscripts utilizing custom algorithms or software that are central to the research but not yet described in published literature, software must be made available to editors/reviewers. We strongly encourage code deposition in a community repository (e.g. GitHub). See the Nature Research guidelines for submitting code & software for further information.

Data

Policy information about availability of data

All manuscripts must include a data availability statement. This statement should provide the following information, where applicable:

- Accession codes, unique identifiers, or web links for publicly available datasets
- A list of figures that have associated raw data
- A description of any restrictions on data availability

Raw RNA-Seq datasets are available at the Sequence Read Archive (BioProject accession number PRJNA542628 [https://www.ncbi.nlm.nih.gov/bioproject/PRJNA542628/]). Differential expression data used in all main figures can be found in Supplementary Data 1. Results for gene set enrichment analysis on previously published gene panels are in Supplementary Data 2. Entropy values and predictions associated with Figure 4 are in Supplementary Data 3. The previously published RNA-Seq dataset used in Figure 5 is available under the BioProject accession number PRJNA518730 [https://www.ncbi.nlm.nih.gov/sra/?term=PRJNA518730]. Gene homology across species was obtained from PATRIC [https://www.patricbrc.org/], and gene functional annotation for enrichment analysis was obtained from UniProt [https://www.uniprot.org/]. Source data are provided with this paper.

Field-specific reporting

Please select the one below that is the best fit for your research. If you are not sure, read the appropriate sections before making your selection.

☒ Life sciences ☐ Behavioural & social sciences ☐ Ecological, evolutionary & environmental sciences

For a reference copy of the document with all sections, see nature.com/documents/nr-reporting-summary-flat.pdf

Life sciences study design

All studies must disclose on these points even when the disclosure is negative.

Sample size	The sizes of the training and test sets are reported in Supplemental Table 8. Supplemental Table 1 summarizes which experiments belong to which sets for each classifier. The sizes of training and test sets corresponded to an approximate 70:30 split, which is standard in studies that involve statistical learning. For regression analyses where the number of samples (i.e. experiments) were smaller than the number of possible features, crossvalidation analysis and lasso regression were used to avoid overfitting the regression models, which is described in the main text and suppelemental methods in detail.
Data exclusions	No data were excluded in the analyses.
Replication	All RNA-Seq experiments were done using at least 3 biological replicates per treatment. All experimental evolution experiments were carried out as 4 independent populations. For error estimates on fitness predictions, 5-fold crossvalidation was used, as detailed in the main text. High correlation across replicates was observed, confirming successful replication of each experiment.
Randomization	For all crossvalidation experiments, the training and test splits were done randomly. The training and test set split was done based on the order in which data collection was done, which was random.
Blinding	Fitness or MOA outcome in the test set was blinded during predictions, and were revealed after prediction to evaluate performance. During data collection and processing and preliminary analyses were not blinded, because the protocols were defined prior to the experiments and objective outcomes were observed.

Reporting for specific materials, systems and methods

We require information from authors about some types of materials, experimental systems and methods used in many studies. Here, indicate whether each material, system or method listed is relevant to your study. If you are not sure if a list item applies to your research, read the appropriate section before selecting a response.

Materials & experimental systems

n/a	Involved in the study	
☒	☐	Antibodies
☒	☐	Eukaryotic cell lines
☒	☐	Palaeontology
☒	☐	Animals and other organisms
☒	☐	Human research participants
☒	☐	Clinical data

Methods

n/a	Involved in the study	
☒	☐	ChIP-seq
☒	☐	Flow cytometry
☒	☐	MRI-based neuroimaging
